# Oxidative RNA Modifications as an Early Response of Soybean (*Glycine max* L.) Exposed to Copper and Lead

**DOI:** 10.3389/fpls.2021.828620

**Published:** 2022-01-31

**Authors:** Jagna Chmielowska-Bąk, Ekaterina Shcheglova, Konrad Rosik, Nikita Yushin, Inga Zinicovscaia, Joanna Deckert

**Affiliations:** ^1^Department of Plant Ecophysiology, Faculty of Biology, Institute of Experimental Biology, Adam Mickiewicz University, Poznań, Poland; ^2^Frank Laboratory of Neutron Physics, Joint Institute for Nuclear Research, Dubna, Russia; ^3^Horia Hulubei National Institute for R&D in Physics and Nuclear Engineering, Magurele, Romania

**Keywords:** 8-hydroxyguanosine, 8-OHG, RNA modifications, oxidative stress, ROS, metal stress, epitranscriptomics

## Abstract

Plant exposure to metals is associated with the accumulation of reactive oxygen species, which mediate the oxidation of various molecules including lipids, proteins, and nucleic acids. The aim of the present study is the evaluation of the impact of short-term Cu and Pb treatment on oxidative events in the roots of soybean seedlings, with special emphasis on RNA oxidation. The results show that an increase in total RNA oxidative modification, 8-hydroxyguanosine (8-OHG), constitutes a very early response to both applied metals, observed already within the first hour of treatment. Exposure to Cu and Pb resulted also in the increase in superoxide anion and hydrogen peroxide levels and intensified lipid peroxidation. However, these responses were most prominent after longer treatment times. On the other hand, no changes were observed in the level of protein carbonylation. It can be concluded that 8-OHG enrichment in total RNA constitutes one of the earliest reactions to metals, which precedes the symptoms of oxidative stress.

## Introduction

Metals, including copper (Cu) and lead (Pb) constitute one of the most important environmental pollutants. High Cu and Pb levels were evidenced in soil samples collected in distinct parts of the world including industrious regions in Europe (Tóth et al., 2016), urban soils in China (Tong et al., 2020), and several regions in Africa (Yabe et al., 2010). Importantly, metals might be taken up from the environment by crop plants and in this way enter the human food chain. Elevated levels of Cu and Pb were noted in vegetables grown in Bangladesh, Brazil, China, India, Nigeria, Pakistan, and Saudi Arabia (reviewed in Sharma and Nagpal, 2020).

Copper is naturally found in the soil usually at the level of 2–110 mg/kg. However, its concentration in the environment might significantly increase due to anthropogenic activities such as mining, waste incarnation, traffic, and application of fertilizers (reviewed in Kumar and Prasad, 2018). In organisms, it can have contrasting effects. On the one hand, Cu constitutes an important component of metalloproteins, including plastocyanins, cytochrome c oxidase, polyphenol oxidases, nitrate reductase, CuZn superoxide dismutase, and ethylene receptors. Through the action of these proteins, Cu is engaged in the processes of photosynthesis, respiration, cell wall metabolism, stress signaling, and defense mechanisms (MacPherson and Murphy, 2007; Pérez-Henarejos et al., 2015, Mir et al., 2021). Thus, Cu deficiency alerts normal plant functioning. On the other hand, excess of this metal also negatively affects plants. High Cu concentrations lead to hampered germination, reduced growth, alerted plant morphology and mineral homeostasis, chlorosis, decreased photosynthesis efficiency, and oxidative stress reflected by accumulation of reactive oxygen species (ROS) and intensified lipid peroxidation (reviewed in Mir et al., 2021).

In contrast to Cu, lead is a non-essential element characterized by high toxicity. It is located on the second position on the Agency for Toxic Substances and Disease Register (ASTDR) Substances Priority List.^1^ It has been reported that exposure to high Pb concentrations inhibits germination of seeds and growth of young seedlings in various plant species. Also in developed plants, Pb hampers growth and affects morphology. This effect is observed in particular in the case of roots, most probably due to limited Pb mobility. Similar to other metals, Pb competes with the uptake of nutrients, which results in nutrient imbalance. This metal has been shown to inhibit the activity of several enzymes including δ-aminolevulinate acid dehydratase (ALAD) engaged in chlorophyll biosynthesis, ribulose-1,5 bisphosphate (Rubisco), and phosphoenolpyruvate carboxylase (PEPC) – key enzymes in CO_2_ fixation, glutamine synthase, and nitrate reductase involved in nitrogen metabolism, proteases, deoxyribonucleases, and ribonucleases. A common feature of Pb-treated plants includes a decrease in photosynthesis efficiency and alerted water status (reviewed in Kumar and Prasad, 2018, Zulfiqar et al., 2019).

One of the most common reactions of plants to environmental stresses, including the excess of metals, is increased production of ROS - hydrogen peroxide (H_2_O_2_), superoxide anion (O_2_^.–^), singlet oxygen (O_2_^1^), and hydroxyl radical (⋅OH). The role of ROS in plant stress response is ambiguous. These highly reactive molecules oxidate cellular compounds, which might lead to membrane damage, altered structure and functions of proteins, and DNA mutations. However, ROS are also engaged in signaling events, either directly or through oxidation of other molecules (reviewed in Waszczak et al., 2018; Castro et al., 2021). There is increasing evidence that also oxidatively modified transcripts might be engaged in the regulation of gene expression (reviewed in Chmielowska-Bąk et al., 2019). Studies on sunflower and wheat showed that transcript enrichment with 8-hydroxyguanosine (8-OHG), the most extensive oxidatively modified ribonucleotide base, is associated with alleviation of seeds dormancy. The studies also showed that 8-OHG formation is limited to certain transcripts (Bazin et al., 2011; Gao et al., 2013). Experiments on reconstituted bacterial systems revealed that 8-OHG formation hampers the process of translation (Simms et al., 2014). These findings indicated that 8-OHG formation is a selective process, which leads to decreased levels of specific proteins.

Despite a possibly important role of transcript oxidation in post-transcriptional regulation of gene expression, so far little attention has been paid to this topic, in particular concerning plant stress response. In our previous study, we have shown that Cd induces transcript oxidation, which was reflected by augmented levels of 8-OHG. An increase in the 8-OHG level was a rapid response observed after 3 h of Cd treatment. On the other hand, ROS over-accumulation and increased lipid peroxidation have been noted only after 24 h of treatment. The results indicated that 8-OHG formation precedes the symptoms of oxidative stress (Chmielowska-Bąk et al., 2018).

In the present research study, we aimed to verify the hypothesis that early 8-OHG formation in total RNA is induced also by other metals. To verify the proposed hypothesis, the 8-OHG level has been quantified in total RNA in the roots of soybean seedlings exposed to Cu and Pb for 1, 3, and 24 h. To embed transcript oxidation within the general oxidative response of soybean seedlings, ROS accumulation and intensity of lipid peroxidation and protein carbonylation were also assessed.

## Materials and Methods

### Cultivation and Treatment Procedures

The experiments have been carried out on soybean as a model plant. Soybean seeds were kindly supplied by the Department of Genetics and Plant Breeding at the Poznań University of Life Sciences. The cultivation has been carried out as described earlier (Chmielowska-Bąk et al., 2018). In short, soybean seeds were surface-sterilized for 5 min with 75% ethanol and 10 min with 1% sodium hypochlorite. The sterilized seeds were washed for 30 min under running water and imbibed for 3–4 h in tap water. The seeds were placed in Petri dishes of 300 mm diameter (in the case of cultivation in sterile conditions) or plastic trays lined with two layers of lignin and one layer of blotting paper, watered, covered with aluminum foil, and placed in a growing chamber in the dark at temperature set for 21–22°C.

After 48 h of germination, the seedlings selected in relation to similar roots length were transferred to Petri dishes of 100 mm of diameter, wherein the roots were placed in the cut-out holes between two layers of lignin. The seedlings were treated for 1, 3, or 24 h with the following solutions: distilled water (control), CuSO_4_ solution containing Cu at the concentration 15 and 30 mg/l and PbCl_2_ solution with Pb at the concentration 300 and 600 mg/l. The concentration of Cu has been chosen on the basis of previous studies conducted on soybean (Chmielowska et al., 2008). The concentration of Pb has been chosen on the basis of root tolerance index – the higher concentration (600 mg/l) inhibited roots growth by 50%. After particular treatment time, the roots of the seedlings were cut off on ice and immediately analyzed (in the case of the measurements of superoxide anion and lipid peroxidation), dried for 72 h at 55°C (for elemental analysis) or frozen in liquid nitrogen and stored at −80°C (for RNA isolation, measurements of H_2_O_2_ level, and determination of protein carbonylation).

### Quantification of Cu and Pb Uptake

The content of Cu and Pb in samples was determined using atomic absorption spectrometer iCE 3400 AAS series with electrothermal atomization (Thermo Fisher Scientific, Waltham, MA, United States). The calibration solutions were prepared from standard solutions for atomic absorption spectrometry with metal ion concentrations of 1 g/L (Merck, Darmstadt, Germany).

Before analysis, the samples were subjected to digestion in a microwave digestion system (Mars; CEM, Metthews, United States). Soybean samples were placed in a Teflon vessel and treated with 2 mL of concentrated HNO_3_ (analytical grade, Merck, Darmstadt, Germany) and 1 mL of H_2_O_2_ (analytical grade, Merck, Darmstadt, Germany). Digestion was performed in two steps: (1) ramp: temperature 180°C, time 15 min, power 400 W, and pressure 20 bar; (2) hold: temperature 160°C, time 10 min, power 400 W, and pressure 20 bar. Then digests were quantitatively transferred to 50-mL calibrated flasks and made up to the volume with bi-distilled water.

Quality control of the measurements was ensured using two reference materials from the National Institute of Standards and Technology: 1570a (Trace Elements in Spinach Leaves) and NIST SRM 1575a (Trace Elements in Pine Needles). The difference between the measured and certified values did not exceed 3%. The measurements were carried out in three independent experimental repetitions each consisting of 10 seedlings.

### RNA Isolation

RNA isolation has been carried out using EXTRAzol reagent (EM30, Blirt, Gdańsk, Poland) according to the manufacturer’s manual. In short, 1 ml of EXTRAzol was added to the samples. Thereafter, the samples were homogenized for 3 min at 25 rounds/min using TissueLyser II (Qiagen, Hilden, Germany) and stainless-steel beads (Cat No./ID: 69989, Qiagen). The samples were incubated for 20 min at room temperature (RT), supplemented with 200 μl of chloroform (Sigma–Aldrich, Saint Louis, United States, 32211), thoroughly mixed, incubated for 15 min at RT, and centrifuged for 20 min at 4°C by 12,000 rpm. The aqueous phase was transferred to new Eppendorf tubes, supplemented with 500 μl of isopropyl alcohol (BioShop, Burlington, Canada, ISO920.500), thoroughly mixed, incubated for 15 min at room temperature (RT), and centrifuged for 15 min at 4°C by 12,000 rpm. The aqueous phase was discarded, the pellet was washed with 1 ml of 75% ethanol (POCH Basic, Gliwice, Poland, BA6480111) and centrifuged for 5 min at 4°C by 7,600 rpm. The ethanol was discarded, the pellet dried for 5 min at RT, and dissolved in RNase/DNase water (BioShop, WAT333.500). Procedures were carried out in sterile conditions.

### Quantification of the Level of 8-OHG

The level of 8-OHG has been assessed using OxiSelect Oxidative RNA Damage ELISA-8OHG Quantification Kit (Cell Biolabs, San Diego, United States, STA-325). Before the procedure, 10 μg of total RNA was digested at 37°C with 20 U of Nuclease S1 (Bio Shop Canada, Burlington, NUC333.50) for 2 h and with 10 U of alkaline phosphatase from bovine intestinal mucosa (Sigma–Aldrich, P6774-2KU) for further 1 h. The subsequent steps were carried out according to the manufacturer’s instructions. The absorbance at λ = 450 nm was measured on İMARK Microplate Reader (Bio-Rad, Hercules, United States) and analyzed with İMARK software. The analyses were performed in 3–4 biological repetitions, each consisting of 10 roots.

### Assessment of the Level of Reactive Oxygen Species

The level of superoxide anion has been measured according to Doke (1983). The roots of soybean seedling were cut off on ice and incubated in the dark at RT in a mixture containing 0.05% NBT (Serva, Heidelberg, Germnay, 30550.03) with 0.1 mM EDTA and 10 mM NaN_3_ (Sigma–Aldrich, 71290) dissolved in 0.05 M potassium phosphate buffer at pH = 7.8. The roots were then weighted and the remaining mixture was incubated for 15 min at 85°C and cooled on ice. The absorbance of the mixture was measured at λ = 580 nm. The analyses were performed in 3–4 biological repetitions, each consisting of 10 roots.

The level of hydrogen peroxide has been measured using Hydrogen Peroxide Assay Kit (Abcam, Cambridge, United Kingdom, Ab 102500) based on OxiRed Probe. The previously cut-off roots of soybean seedlings (approximately 150 mg), frozen in liquid nitrogen and stored at −80°C were homogenized in cooled mortars in liquid nitrogen. The homogenized samples were transferred to Eppendorf tubes supplemented with 500 μl of Assay Buffer and centrifuged for 15 min at 4°C at 13,000 rcf. Afterward, 50 μl of supernatant was added to wells and supplemented with 50 μl of Reaction Mixture provided by the manufacturer. For negative probe Assay, buffer has been used instead of the supernatant. Due to the fast color change, the absorbance has been measured immediately by λ = 570 nm. Each sample has been measured in two technical repetitions. The analyses were performed in 3–6 biological repetitions, each consisting of 10 roots.

### Assessment of Lipid Peroxidation

Lipid peroxidation has been assessed on the basis of the amount of thiobarbituric reactive substances (TBARS) according to Cuypers et al. (2011) with small modifications (Cuypers et al., 2011). Approximately 200 mg of roots were cut off on ice and homogenized in 2 ml of 10% trichloroacetic acid (TCA) (Sigma–Aldrich, TO699). Then the samples were centrifuged for 20 min at 4°C by 12,000 rpm. The supernatant was transferred to a glass tube, supplemented with 4 ml of 0.5% thiobarbituric acid (TBA; Sigma–Aldrich, T5500) dissolved in 10% (TCA; Sigma–Aldrich, T0699) and incubated for 30 min at 95°C. After heating, the samples were cooled and their absorbance was measured at λ = 532 nm and corrected for unspecific absorbance at λ = 600 nm. The measurements were performed in 4–6 biological repetitions, each consisting of 10 roots.

### Quantification of Protein Carbonylation

Protein carbonylation has been assessed with the use of Protein Carbonyl Content Assay Kit (Sigma–Aldrich, MAK094). Approximately 150 mg of soybean seedlings roots were homogenized in cooled mortars in 500 μl of water free from RNAses, DNAses, and Proteinases (BioShop, WAT333.500). The samples were transferred to new Eppendorf tubes and centrifuged for 5 min by 13,000 g at 4°C. Then, 10 μl of 10% Streptozocin has been added, the samples were incubated for 15 min at 20–22°C (RT) and centrifuged for 5 min by 13,000 g at 4°C. The supernatant was transferred to new Eppendorf tubes and supplemented with 100 μl of provided 2,4-dinitrophenylhydrazine. The samples were incubated for 10 min at 20–22°C (RT), supplemented with 30 μl of 100% provided TCA, vortexed, incubated for 5 min in ice, and centrifuged for 2 min by 13,000 g at 4°C. The supernatant was transferred to new Eppendorf tubes and was supplemented with 500 μl of ice-cold acetone (BA8760114). Next, the samples were sonicated for 2 min, incubated for 5 min in −20°C, and centrifuged for 2 min by 13,000 g at 4°C. The supernatant has been removed, the pellet was dissolved in 200 μl of provided guanidine solution and sonicated for 30 s. The samples were transferred to a microplate (100 μl per well) and the absorbance has been measured by the λ = 375 nm on Synergy LX microplate reader (BioTek, Wnooski, United States). Pure guanidine solution has been used as the negative control. Each sample was assayed in two technical replications.

The protein level was measured with the use of QuantiPro BCA Assay Kit (Sigma–Aldrich QPBCA) according to the manufacturer’s manual. Briefly, 50–100 μl of supernatant was transferred to microplate wells and supplemented with DNAase/RNase/proteinase-free water (BioShop, WAT333.500) to the total volume of 150 μl. Thereafter, each well has been supplemented with 150 μl of provided Working Reagent. Due to rapid color change, the absorbance of the samples has been measured after 30 min of incubation at 37°C by the λ = 562 nm. Each sample was measured in two technical repetitions.

The level of carbonyl groups has been calculated according to the formula provided in the Protein Carbonyl Content Assay Kit manual:


$$\mathrm{CP}={\left(A_{375}/6.364\right)}^{*}100/\mathrm{mg}\mathrm{of}{\mathrm{protein}}^{*}1000,$$

CP – nmol carbonyl/mg protein.

The analyses were performed in 4–6 biological repetitions, each consisting of 10 roots.

### Statistical Analysis

The statistically significant differences between data obtained from control and metal-treated plants have been calculated on the basis of a one-way ANOVA test by the *p* = 0.05 using Statistica 13.3 software (StatSoft, Tulsa, United States). The statistically significant different results in relation to the control have been marked on the graphs with different letters (in the case of metal uptake) or asterisk (*).

## Results

**FIGURE 1:**
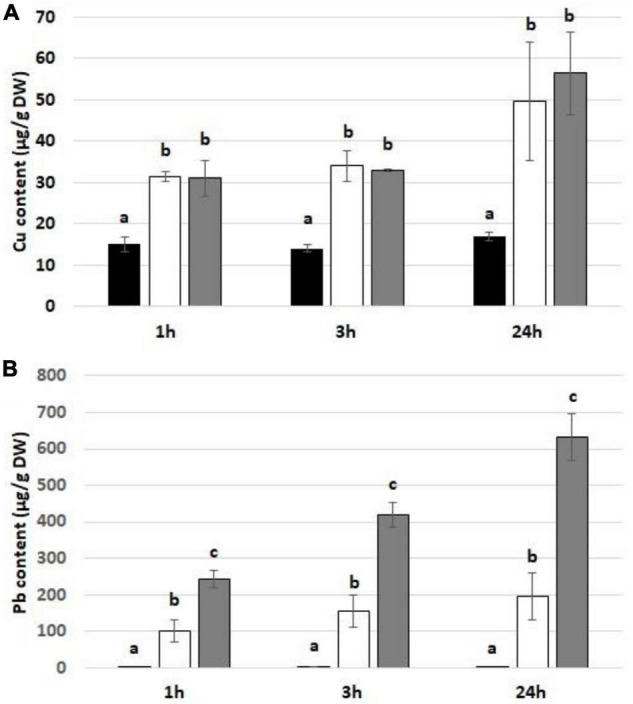
The level of Cu **(A)** and Pb **(B)** in soybean seedlings exposed to the respective metal for 1, 3, and 24 h in control seedlings (black bars) and seedlings exposed to Cu at the concentration 15 mg/l or Pb at the concentration 300 mg/l (white bars) and Cu at the concentration 30 mg/l or Pb at the concentration 600 mg/l (gray bars). The statistically significant differences in relation to the control are marked with different letters (a–c). The results are means of three experimental repetitions ± standard error.

Treatment with Cu and Pb resulted in significant uptake of these metals by soybean seedlings (Figure 1). The level of Cu was significantly higher in metal-treated seedlings, although it did not vary between various concentrations and treatment times (Figure 1A). In the case of Pb, metal uptake was increasing with the applied concentration and treatment duration (Figure 1B).

**FIGURE 2:**
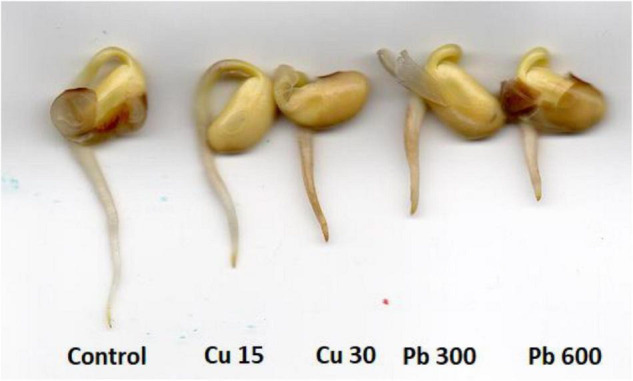
The morphology of control soybean seedlings and soybean seedlings subjected for 24 h to CuSO_4_ solution with Cu at concentration 15 and 30 mg/l (Cu 15 and Cu 30, respectively) and Pb solution with PbCl_2_ at the concentration 300 and 600 mg/l (Pb 300 and Pb 600, respectively).

No visible effects on the seedlings’ morphology were observed after 1 and 3 h of metal treatment (data not shown). However, exposure to Cu and Pb for 24 h resulted in roots shortening and browning (Figure 2).

**FIGURE 3:**
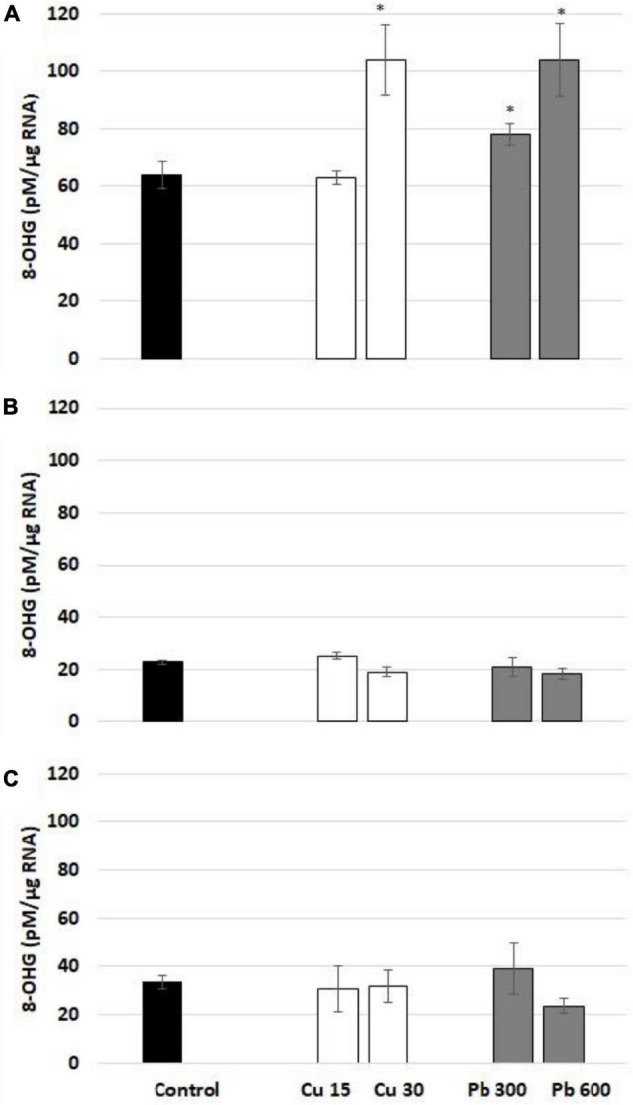
The level of 8-OHG in RNA after 1 **(A)**, 3 **(B)**, and 24 h **(C)**. In the roots of control (black bar) soybean seedlings and the roots of soybean seedling exposed to Cu at concentrations 15 and 30 mg/l (white bars) and Pb at concentrations 300 and 600 mg/l (gray bars). The results are means of 3–4 experimental repetitions ± standard error. The statistically significant differences in relation to the control are marked with asterisk (*).

Short-term exposure to Cu and Pb resulted in a significant increase in 8-OHG level in total RNA isolated from the roots of soybean seedlings (Figure 3). The observed stimulation of 8-OHG formation was a rapid process observed only after the first hour of metal treatment. In the case of Cu, the increase in 8-OHG level was noted in response to the higher concentration (30 mg/l). In the case of Pb, both applied concentrations (300 and 600 mg/l) induced 8-OHG formation, although the induction was much stronger in response to the treatment with Pb at the highest concentration.

**FIGURE 4:**
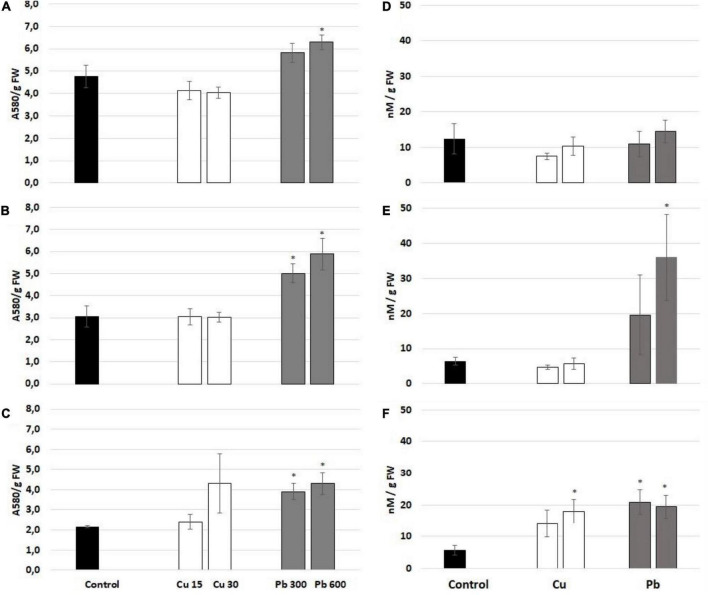
The level of superoxide anion measured after 1 **(A)**, 3 **(B)**, and 24 h **(C)** and hydrogen peroxide assessed after 1 **(D)**, 3 **(E)**, and 24 h **(F)** in the roots of control (black bar) soybean seedlings and the roots of soybean seedling exposed to Cu at concentrations 15 and 30 mg/l (white bars) and Pb at the concentration 300 and 600 mg/l (gray bars). The results are means of 3–6 experimental repetitions ± standard error. The statistically significant differences in relation to the control are marked with asterisk (*).

To evaluate the general oxidative status of the roots of soybean, the seedlings exposed to Cu or Pb, the level of ROS (superoxide anion and hydrogen peroxide), lipid peroxidation (reflected by TBARS level), and protein carbonylation were assessed. Exposure to Cu had no significant effect on the level of superoxide anion (Figures 4A–C). On the contrary, Pb treatment increased the level of superoxide anion observed after all treatment times (Figures 4A–C). The levels of hydrogen peroxide were increased in response to 3 and 24 h long treatment with Pb and 24 h long treatment with Cu (Figures 4D–F).

**FIGURE 5:**
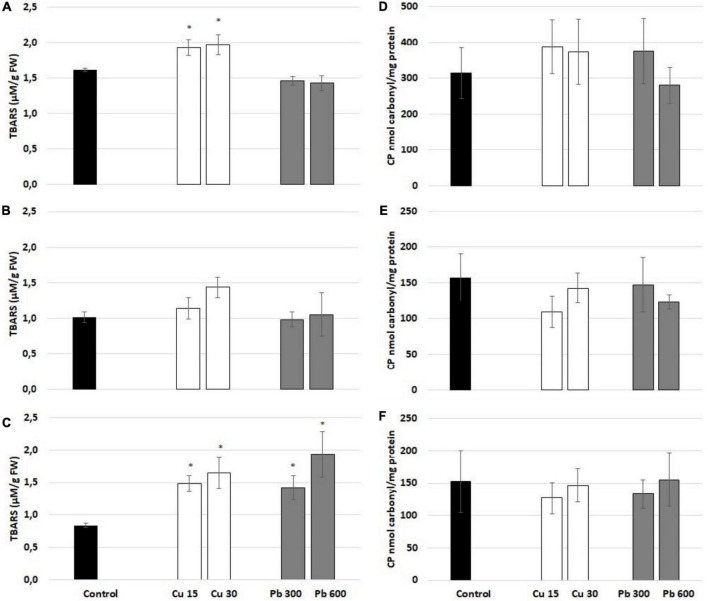
Lipid peroxidation reflected by the level of thiobarbituric acid reactive substances (TBARS) assessed after 1 **(A)**, 3 **(B)**, and 24 h **(C)** and protein carbonylation assessed after 1 **(D)**, 3 **(E)**, and 24 h **(F)** in the roots of control (black bar) soybean seedlings and the roots of soybean seedling exposed to Cu at concentrations 15 and 30 mg/l (white bars) and Pb at concentrations 300 and 600 mg/l (gray bars). The results are means of 4–6 experimental repetitions ± standard error. The statistically significant differences in relation to the control are marked with asterisk (*).

The pattern of lipid peroxidation was metal- and time-dependent (Figures 5A–C). The levels of lipid peroxidation marker, TBARS, were elevated in the roots of soybean seedlings treated with Cu for 1 and 24 h (Figures 5A,C). In the case of Pb, lipid peroxidation was increased only after 24 h of treatment (Figure 5C). Protein carbonylation was unaffected by Cu and Pb at any of the applied concentrations and treatment times (Figures 5D–F).

## Discussion

Excess of metals, such as Cu and Pb exhibits toxic effects and leads to severe disturbances in plants homeostasis (reviewed in Kumar and Prasad, 2018, Zulfiqar et al., 2019; Mir et al., 2021). To activate defense mechanisms and survive unfavorable conditions, plants need to transduce stress signals and modulate gene expression. ROS are recognized as important signaling elements. Their signaling functions might depend on oxidation of thiol groups in certain proteins, interactions with other signaling elements, or generation of molecules that might themselves transduce the signal (Chmielowska-Bąk et al., 2015; Waszczak et al., 2018, Castro et al., 2021).

Several premises indicate that signaling and/or gene regulatory function might be exerted also by oxidized transcripts. First, it has been shown that the formation of the most frequent RNA oxidative modification, 8-OHG, is a selective process limited to a specific set of mRNA species. Second, it has been demonstrated that 8-OHG enrichment disturbs translation and leads to a decrease in the level of encoded proteins (Shan et al., 2003; Chang et al., 2008, Bazin et al., 2011; Gao et al., 2013). Thus, selective oxidation of transcripts leads to decreased expression of certain proteins. This process has been shown to play a role in the development of neurodegenerative diseases in humans (Shan et al., 2003, 2007, Chang et al., 2008) and in the alleviation of seed dormancy in plants (Bazin et al., 2011; Gao et al., 2013). However, so far the role of oxidatively modified transcripts in plants’ response remains unexplored.

In the present study, we show that 8-OHG formation in total RNA is induced by exposure to Cu and Pb in the roots of soybean seedlings. This response was observed only after the first-hour of treatment indicating that RNA oxidation constitutes a very rapid reaction to the metals (Figure 3). Similar results were observed in soybean seedlings exposed to Cd. In this case, elevated levels of 8-OHG were noted after 3 h of exposure to the metal. On the other hand, the markers of oxidative stress such as lipid peroxidation or accumulation of abasic sites in transcripts were evidenced only after 24 h of Cd stress (Chmielowska-Bąk et al., 2018). These results indicate that 8-OHG formation in transcripts is an early and universal response to metals.

It is not clear which ROS species could be responsible for the rapid 8-OHG formation. Exposure to Pb resulted in elevated superoxide anion levels in all treatment times including 1 h of exposure. Accumulation of hydrogen peroxide occurred later - after 3 and 24 h. On the other hand, treatment with Cu did not affect the level of superoxide anion in any of the treatment times, while Cu-dependent hydrogen peroxide accumulation has been noted only after 24 h (Figure 4). Observed oxidation of the total RNA in soybean seedlings roots may be mediated by a short-time spike/spikes in ROS production occurring in even earlier stages of metal treatment. The studies on *Arabidopsis* plants showed that ROS can be induced within minutes or even seconds after wounding (Miller et al., 2009; Gilroy et al., 2014). In the case of metal stress, rapid increase in ROS observed within the first hour of exposure has been noted, for example, in tobacco suspension cells treated with Cd (Garnier et al., 2006), *Arabidopsis thaliana* treated with Cd or Cu (Maksymiec and Krupa, 2006), alfalfa seedlings treated with Cd and Hg (Ortega-Villasante et al., 2007) and barley root tips treated with Cd, Cu, Pb, or Hg (Tamás et al., 2017).

One of the universal responses to prolonged metal exposure is increased lipid peroxidation, which is considered a marker of oxidative stress. In the present study both metals, Cu and Pb induced, lipid peroxidation in seedlings roots, whereas the response was most evident after 24 h of metal treatment (Figure 4). These results are consistent with numerous other reports. Increased TBARS and/or MDA levels in response to Cu were observed among others in wheat seedlings (Gajewska and Skłodowska, 2010), maize hydroponic culture (Ahmed et al., 2021), rice roots (Thounaojam et al., 2012), cucumber (Yusuf et al., 2021), tomato (Cui et al., 2010), and sour orange plants (Giannakoula et al., 2021). Intensified lipid peroxidation in reaction to Pb was evidenced, for example, in rice (Srivastava et al., 2014), pea (Malecka et al., 2009), cotton seedlings (Priyanka et al., 2021), corn leaves (Kisa et al., 2020), and sour orange plants (Giannakoula et al., 2021).

Reactive oxygen species also mediate the oxidation of proteins reflected by protein carbonylation. Elevated levels of carbonyl groups were noted in the roots and shoots of Pb-treated rice (Srivastava et al., 2014), shoots of *Silene vulgaris* exposed to Pb, Zn, and Cd (Muszyńska and Labudda, 2020), roots and shoots of wheat seedlings treated with Cu (Gajewska and Skłodowska, 2010), and sunflower and barley leaves in response to Cu (Demirevska-Kepova et al., 2004; Pena et al., 2008). In the present study, neither Cu nor Pb had an effect on protein carbonylation in the roots of soybean seedlings. This could result from short treatment times as the oxidative status has been analyzed within the first 24 h of metal treatment. In contrast, in all of the above-cited studies the applied treatment times were much longer – the plants were exposed to metals at minimum for 4 days and at maximum for 8 weeks. Thus, protein carbonylation may be a marker of prolonged metal stress.

The results indicate that 8-OHG formation in total RNA in the roots of soybean seedlings is one of the earliest reactions to Cu and Pb exposure and precedes the most pronounced accumulation of oxidative stress markers such as lipid peroxidation or over-production of hydrogen peroxide. Thus, the results indicate that 8-OHG enrichment in total RNA is not a symptom of oxidative stress. We hypothesized that it can be engaged in sensing and/or gene regulatory events. In fact, it has been evidenced earlier that 8-OHG participated in post-transcriptional regulation of gene expression in animal and plant models (Chang et al., 2008; Bazin et al., 2011, Gao et al., 2013). For example, in transgenic mice bearing mutation in *SOD1* gene, which results in induction of amyotrophic lateral sclerosis (ALS), mRNA oxidation also occurred early, in the pre-symptomatic stage of the disease. Importantly, the oxidation has been limited to a certain set of transcripts, and many of the oxidized mRNA species have been implicated in the pathogenesis of ALS. In addition, 8-OHG enrichment in certain transcripts led to the decrease in the level of encoded proteins (Chang et al., 2008). Similarly, breakage of wheat seeds dormancy was associated with 8-OHG formation in certain sets of transcripts including α-amylase/trypsin inhibitor and starch synthase. The oxidation of transcripts was correlated with a decrease in the level of encoded proteins. Thus, it seems that oxidation of targeted transcripts leads to a decrease in the level of the inhibitors of storage material mobilization and in this way facilitated the germination (Gao et al., 2013). It is therefore possible that the observed early 8-OHG formation participates in metal-dependent gene regulation and possible activation of defense mechanisms. However, this is a hypothesis that would need further complex verification.

The transient nature of the 8-OHG formation, which was induced after 1 h of metal treatment and reached the levels observed in control after 3 and 24 h, could be a result of degradation of oxidized transcripts. It has been noted in the HeLa cell line that the level of 8-OHG decreased by 50% within the first hour of oxidative stress. It has been also reported that 8-OHG-enriched transcripts are recognized by proteins engaged in RNA metabolism such as polynucleotide phosphorylase (PNPase) in *E. coli* and YB-1 protein in humans (Li et al., 2006). Recently it has been proposed that in HeLa cells the transcripts bearing limited 8-OHG nucleotides are recognized by AUF1 protein and directed for degradation. On the other hand, heavily oxidized transcripts are bound by PCBP1 protein, which activates mechanisms leading to cell apoptosis (Ishii and Sekiguchi, 2019).

## Conclusions

**FIGURE 6:**
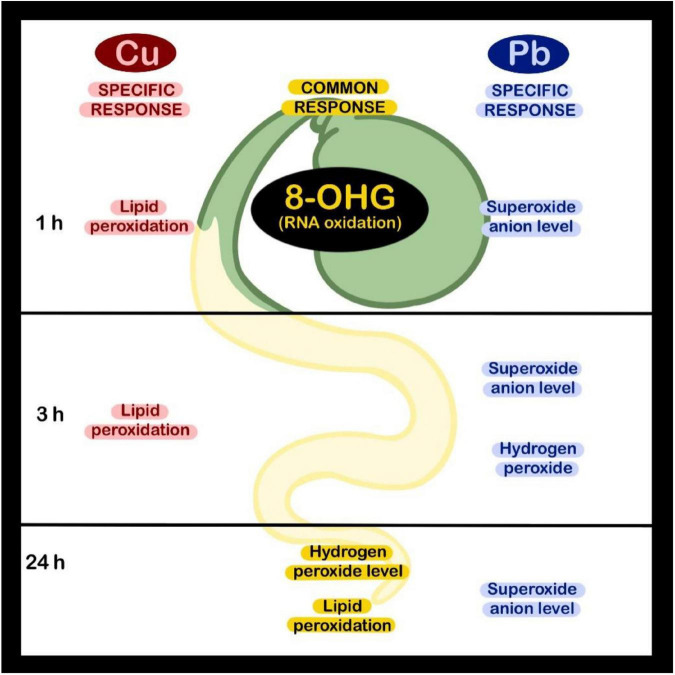
Presentation of the elements of oxidative response induced in soybean seedlings in reaction to short-term Cu or Pb treatment.

In conclusion, the oxidative response observed in the roots of soybean seedlings exposed to Cu and Pb was dependent on the applied metal, concentration, and treatment duration (summarized in Figure 6). A common response to both metals observed after 1 h of treatment was increased in the level of 8-OHG in total RNA. In turn, ROS accumulation and lipid peroxidation in the seedlings’ roots were most prominent after longer exposure to Cu and Pb. Similar results obtained in soybean seedlings treated with Cd (Chmielowska-Bąk et al., 2018) indicate that early 8-OHG formation constitutes a universal reaction to metals. The exact role of such rapid, metal-dependent 8-OHG induction in plant stress response would need further elucidation.

## Data Availability Statement

The raw data supporting the conclusions of this article will be made available by the authors, without undue reservation.

## Author Contributions

JC-B, IZ, and JD: conceptualization, methodology, and supervision. IZ and NY: quantification of metal uptake. JC-B, ES, and KR: measurement of 8-OHG levels. JC-B and KR: assessment of ROS levels and protein carbonylation. ES and JC-B: assessment of lipid peroxidation. JC-B: data curation and writing—original draft preparation. JC-B, JD, IZ, and ES: writing—review and editing. JC-B and IZ: project administration and funding acquisition. All authors have read and agreed to the published version of the manuscript.

## Conflict of Interest

The authors declare that the research was conducted in the absence of any commercial or financial relationships that could be construed as a potential conflict of interest.

## Publisher’s Note

All claims expressed in this article are solely those of the authors and do not necessarily represent those of their affiliated organizations, or those of the publisher, the editors and the reviewers. Any product that may be evaluated in this article, or claim that may be made by its manufacturer, is not guaranteed or endorsed by the publisher.

## Footnotes

1
https://www.atsdr.cdc.gov/spl/index.html
